# Extracellular vesicles promote migration despite BRAF inhibitor treatment in malignant melanoma cells

**DOI:** 10.1186/s12964-024-01660-4

**Published:** 2024-05-22

**Authors:** Afrodité Németh, Gréta L. Bányai, Nikolett K. Dobos, Tamás Kós, Anikó Gaál, Zoltán Varga, Edit I. Buzás, Delaram Khamari, Magdolna Dank, István Takács, A. Marcell Szász, Tamás Garay

**Affiliations:** 1 Faculty of Information Technology and Bionics, Pázmány Péter Catholic University, Budapest, Hungary; 2 Institute of Materials and Environmental Chemistry; Biological Nanochemistry Research Group, HUN-REN Research Centre for Natural Sciences, Budapest, Hungary; 3 Department of Genetics, Cell- and Immunobiology, Semmelweis University, Budapest, Hungary; 4 ELKH-SE Translational Extracellular Vesicle Research Group, Budapest, Hungary; 5 HCEMM-SE Extracellular Vesicle Research Group, Budapest, Hungary; 6 Department of Internal Medicine and Oncology, Division of Oncology, Semmelweis University, Budapest, Hungary

**Keywords:** Extracellular vesicles, Melanoma, Vemurafenib, Dabrafenib, Trametinib, Single cell tracking, Cell migration

## Abstract

Extracellular vesicles (EVs) constitute a vital component of intercellular communication, exerting significant influence on metastasis formation and drug resistance mechanisms. Malignant melanoma (MM) is one of the deadliest forms of skin cancers, because of its high metastatic potential and often acquired resistance to oncotherapies. The prevalence of BRAF mutations in MM underscores the importance of BRAF-targeted therapies, such as vemurafenib and dabrafenib, alone or in combination with the MEK inhibitor, trametinib. This study aimed to elucidate the involvement of EVs in MM progression and ascertain whether EV-mediated metastasis promotion persists during single agent BRAF (vemurafenib, dabrafenib), or MEK (trametinib) and combined BRAF/MEK (dabrafenib/trametinib) inhibition.

Using five pairs of syngeneic melanoma cell lines, we assessed the impact of EVs – isolated from their respective supernatants – on melanoma cell proliferation and migration. Cell viability and spheroid growth assays were employed to evaluate proliferation, while migration was analyzed through mean squared displacement (MSD) and total traveled distance (TTD) measurements derived from video microscopy and single-cell tracking.

Our results indicate that while EV treatments had remarkable promoting effect on cell migration, they exerted only a modest effect on cell proliferation and spheroid growth. Notably, EVs demonstrated the ability to mitigate the inhibitory effects of BRAF inhibitors, albeit they were ineffective against a MEK inhibitor and the combination of BRAF/MEK inhibitors. In summary, our findings contribute to the understanding of the intricate role played by EVs in tumor progression, metastasis, and drug resistance in MM.

**Supplementary Information:**

The online version contains supplementary material available at 10.1186/s12964-024-01660-4.

## Introduction

Extracellular vesicles (EVs) are small lipid-bound particles, containing a diverse array of macromolecules, ubiquitously present in all body fluids. These particles play pivotal roles in both local and systematic communications, with growing evidence suggesting their utility as prognostic and predictive biomarkers [1]. Notably, elevated plasma EV levels have been associated with adverse outcomes in non-small cell lung [2], colon [3], head and neck cancer [4] or melanoma [5]. Of note, in patients with oral squamous cell carcinoma, a reduction in plasma EV levels has been observed following tumor resection [6], underscoring their potential as dynamic markers of disease burden. In addition, elevated level of plasma EV-associated proteins have been correlated with the occurrence of brain metastasis [7]. In alignment with clinical findings, experimental studies in murine models have further elucidated the contributory role of EVs in metastasis, particularly in highly metastatic melanoma, where EVs have been shown to facilitate metastatic colonization and dissemination [5]. Possible reason why EVs promote migration could be that EVs could transfer integrins [8] and they can promote cell adhesion [9]. Moreover, EVs can affect the phosphorylation of FAK, AKT, and ERK1/2 [10] and transfer mRNAs [11] and miRNAs [12] involved in migration and metastasis formation. Proteome analysis of metastatic melanoma cell lines-derived EVs’ utilizing KEGG (Kyoto Encyclopedia of Genes and Genomes), BBID (Biological Biochemical Image Database), and Biocarta databases showed that EVs are enriched in proteins involved in the regulation of actin cytoskeleton and focal adhesion [13]. Thus, EVs are considered to play a determining role in the process of metastasis formation [14, 15].

Furthermore, they can influence the tumor microenvironment by activating normal human fibroblasts [16] or inactivating macrophages and reprograming the secretory profile of monocytes [17]. Additionally, exosomes derived from melanoma cells have been shown to promote tumor cell proliferation and inhibit apoptosis [18]. Furthermore, EVs modulate the metastatic potential of recipient cells, influencing migratory capacity [19], invasiveness [20], anchorage-independent cell growth [21] and the formation of pre-metastatic niches [22]. Notably, EV populations exhibit heterogeneity across cells with different degrees of malignancy [13], moreover, metastatic tumor-derived EVs augment the metastatic potential of the less aggressive counterparts [11].

Metastasis formation is the primary cause of cancer-related deaths [23], thus the impact of extracellular vesicles (EVs) on metastasis emphasizes their potential significance in the management of metastatic malignancies. One of the most metastatic tumors is melanoma [24, 25] with a 5-year survival of over 90% in patients with a localized tumor and only 16% in metastatic cases [26]. Approximately 40-60% of melanoma cases harbor V600E mutation in BRAF, resulting in constitutive activation of mitogen-activated protein kinase (MAPK) signaling pathway [27–29].

In clinics, one of the potent BRAF-targeting inhibitors is vemurafenib [30–33] and the more recently applied dabrafenib [34, 35]. In preclinical experiments, vemurafenib and dabrafenib increase cell death [36–38], decrease proliferation [39–42] and affect cell migration [43–47]. Furthermore, exposure to these inhibitors can modulate the cargo of released EVs’ [48, 49].

Unfortunately, after the first sensitive period, relapse, and resistance to these BRAF inhibitors are observed in most melanoma cases [34, 35]. In addition, patients treated with vemurafenib frequently showed a distinct increase in the number of brain metastases [50–52]. Resistance can occur with several mechanisms, such as genetic, epigenetic and/or transcriptomic changes [53–55], alterations in EV trafficking is also implicated in BRAF inhibitor resistance [56, 57]. Persistent MEK phosphorylation is also observed under BRAF inhibition [58], prompting the adoption of BRAF/MEK inhibitor combinations [59–61]. This combination of dabrafenib and the MEK inhibitor trametinib improved the patients' progression-free survival, overall survival rates and objective response rates compared to the monotherapies [62]. However, after prominent disease regression, resistance to dabrafenib-trametinib therapy still recurrently develops [55, 63, 64].

Accordingly, we aimed to investigate the role of EVs in cancer progression, employing *in vitro* models of melanoma, utilizing syngeneic pairs of cell lines. Each pair comprised a cell line representing an earlier, less progressed stage of the tumor (e.g. originating from a primary tumor site or isolated from a tumor specimen taken before the start of vemurafenib therapy). In contrast, the other cell line represented a more advanced stage of the disease (e.g. obtained post-relapse, derived from metastatic site or selected as the most tumorigenic sub-clone in mice). EVs produced by the cell lines underwent characterization, with subsequent investigation into their effects on proliferation and migration *in vitro*. Concurrently, our objective was to assess whether treatment with vemurafenib, dabrafenib, and the dabrafenib-trametinib combined treatment could mitigate potential EV-mediated effects.

## Materials and methods

### Cell lines and culturing

Melanoma cells used for the experiments were pairwise originating from the same patient (i.e. representing the same genetic background) and modelling the less and more advanced stages of the given tumor.

Mel Pt-1, Mel Pt-3, Mel Pt-4 pair of cell lines were established and kindly provided by Professor Peter Hersey from the Oncology and Immunology Unit, Calvary Mater Newcastle Hospital and the Kolling Institute, Royal North Shore Hospital, University of Sydney, NSW, Australia [65]. The cell line pairs’ “pre” members were isolated before vemurafenib treatment and “post” members during vemurafenib treatment, each patient was partially responsive to vemurafenib and all cell lines are harboring BRAF V600E mutations [65]. The BRAF V600E mutant WM983A – derived from the patient primary tumor site – and WM983B – originated from the patient’s metastasis – are available at Wistar Institute, Philadelphia, PA, USA, and the A2058 cell line harboring a BRAF V600E mutation at ATCC. M1 cell line was established from A2058 cells as the sub-clone with the greatest tumorigenic potential in immunosuppressed mice [66].

If not indicated other ways all cells were cultured in DMEM (4.5g/L glucose with L-glutamine and Sodium Pyruvate, Capricorn-Scientific) supplemented with 10% fetal bovine serum (FBS, EuroClone) and 1% penicillin-streptomycin-amphotericin (Lonza) at 37 °C in humidified 5% CO_2_ atmosphere.

### Isolation of EVs from cell-culture supernatant:

Prior to harvest the supernatant, cells were grown in all cases in three 75 cm^2^ tissue culture flasks until 50-60% confluency, then cells were washed 2 times with PBS (Capricorn-Scientific) and cultured in DMEM supplemented with 1% EV-depleted FBS (Biowest) for three days. The collected supernatants were centrifuged at 500 g for 5 minutes to remove floating cells and cell debris. Supernatants were stored at -80°C until further use. On the day of the experiments, the frozen supernatants were thawed slowly, centrifuged at 3,000 g for 15 minutes, and filtered through a 0.2 µm syringe filter unit (Sarstedt). Then the filtered samples were subjected to ultracentrifugation (Beckman L7-55 Ultracentrifuge, TYPE 50.2 Ti rotor) at 100,000 g for 1.5 hours at 4 °C and the pellets (EV) were suspended in 300 µl of PBS and used for treatments on the day of the isolation.

### Characterization of EVs

EV samples were first verified using Dynamic Light Scattering (DLS). The isolation was considered successful if the EV sample’s main particle population size was in the range of cell-culture supernatant EVs [67] and the EV depleted supernatant didn’t contain particles larger than 10 nm. Total protein concentration was quantified by Qubit® Protein Assay Kit (Thermo Fisher Scientific) according to the manufacturer’s instructions, and lipid concentration was determined by sulfophosphovanillin lipid assay [68]. Particle concentration and size distribution were evaluated by Nano Particle Tracking Analysis with ZetaView PMX120 NTA instrument (Particle Metrix GmbH, Inning am Am-mersee, Germany). EVs were characterized by flow cytometry (FCM) with a CytoFlex flow cytometer (Beckman Coulter Inc., Brea, California, USA) using commonly used membrane markers. Briefly, the isolated EVs were first attached to 3 μm aldehyde/sulfate latex beads (4% w/v; Thermo Fisher Scientific). The samples were incubated with 1,000-fold diluted latex beads (in PBS) at a 1:1 ratio for 30 minutes at room temperature (RT), 320 rpm on a thermo-shaker. The bead concentration was chosen to be at least 300 EV per latex particle. Then the latex beads were blocked with glycine (100 mM final concentration; Sigma-Aldrich) and BSA (0,5% final concentration; Sigma-Aldrich) for one hour at RT, 320 rpm. Blocking agents were removed by centrifugation at 3,000 g for 5 minutes, and the pellet was resuspended in PBS (5 times the volume of the samples). To fluorescently label EVs on the surface of the beads, the samples were stained using Annexin V-FITC (1:1000; InvitrogenTM BMS500FI-100); EpCAM (1:100; EGP40/1372; GeneTex GTX34694), CD81 (1:100; 1D6; GeneTex GTX75436), CD63 (1:100; MEM-259; GeneTex GTX28219), and CD9 (1:100; MEM-61; GeneTex GTX22215) as primary antibodies and Alexa FluorTM 488-conjugated secondary antibody (1:200; Alexa FluorTM 488 goat anti-mouse IgG; Invitrogen A11029) for 30 minutes at 37°C, 320 rpm. Negative control was prepared by blocking the latex beads with glycine and BSA and incubating them with Annexin V-FITC and the secondary antibody. The samples were measured directly with CytoFlex flow cytometer (Beckman Coulter Inc, Brea, California, USA) and gating of the main latex population (3 μm in diameter) was performed with the CytExpert algorithm. All data is submitted to the EV-TRACK knowledgebase (EV-TRACK ID: EV230027) [69].

### Cell viability (SRB) assays

To explore the impact of Extracellular Vesicles (EVs) on cellular behavior, cells were cultured in a 96-well plate and subsequently treated with EVs (10 μg/ml protein concentration) in DMEM supplemented with 10% EV-depleted FBS for 72 hours. Following treatment, cells were fixed using 10% trichloroacetic acid, then stained with Sulforhodamine B (SRB) after washing and drying the wells. After 15 minutes the stain was discarded, and the cells were washed with 1% acetic acid solution and dried out. The stain was dissolved in 10 mM Tris-HCl, pH 8 and absorbance was determined at 570 nm. Likewise, SRB cell viability assay was used to asses the sensitivity of cell lines to vemurafenib (PLX4032 VWR), dabrafenib (VWR), trametinib (VWR), and the dabrafenib-trametinib combined. The interactions between dabrafenib and trametinib were analysed by CopmuSyn software (ComboSyn Inc), calculating the combination index (CI). CI < 1, CI = 1, CI > 1 represents synergism, additive effects and antagonism, respectively [70].

### Spheroid formation assay:

To establish spheroid cultures, cells were seeded in U-bottom 96 well plates pre-coated with 30 μl of 25 mg/ml poly(2-hydroxyethyl methacrylate) (poly HEMA, Merck) dissolved in 96% ethanol. The plates were then placed on a rocking platform until complete evaporation of the solution (3 days). Cells were seeded at the density of 1,000 cells/well and maintained in DMEM supplemented with 10% EV-depleted FBS and 0.04 mg/ml collagen (Merck). Following seeding, the plates were centrifuged at 2,200 rpm for 10 minutes. After 24h incubation, EV treatment was administered at a final protein concentration of 10 μg/ml. Spheroid growth was monitored by capturing black and white images daily for 7 days and evaluating the pictures using ImageJ. Spheroid size was quantified by assessing the signal intensity (SI), accounting for spheroid area, integrated density (ID), and the median pixel intensity of the respective well, utilizing the following formula. The spheroid size was quantified by asessing the calculated signal intensity (SI) (i.e. darkness) of the spheroids accounting for spheroid area, integrated density (ID), and the median pixel intensity of the respective well using the following formula:

SI = 255 × area − ID − (255 − median) × area

### Cell migration analysis by single-cell tracking

Video-microscopy measurements were used to evaluate cell migration. Plates were prepared and treated as for SRB assays. Vemurafenib was applied in the following concentration: Mel Pt-1 pre/post, Mel Pt-4 pre/post: 10 µM, Mel Pt-3 pre 0.75 µM, Mel Pt-3 post 7.5 µM, WM983A: 0.05 µM, WM983B: 0.25 µM, A2058 and M1: 2.5 µM For Mel Pt-3 pre 20 nM dabrafenib and 2 nM trametinib, for Mel Pt-3 post 50 nM dabrafenib and 5 nM trametinib were applied. After treatment, plates were placed into an inverted phase contrast microscope with an automatic stage and surrounding incubator (Nikon TIE microscope, Prior stage, Oko-Lab incubator) and kept at 37 °C in 5% CO_2_ atmosphere. Time-lapse recording was performed for 24 hours with 10 minutes per frame rate. After pre-processing the images, single-cell tracking and determination of the XY coordinates of the cells were performed with the semiautomatic tracking tool CellTracker [71]. From the cells’ coordinates, two different migration parameters were calculated: total travelled distance (TTD) and mean square displacement (MSD). TTD is calculated as the sum of the distance travelled by the cell between two consecutive images. MSD measures the average square displacement over increasing time intervals between two points [72]. To evaluate the effects of the treatments, first, both MSD and TTD values were averaged for control cells as a function of time. For the averaged-control and for each cell individually, area under the curve (AUC) was computed using MATLAB built-in function trapz() and ∆AUC were calculated as the difference between each individual cells’ AUC and the averaged-control AUC, and ∆AUC values were averaged for each treatment.

### Statistical analysis

Statistical differences between groups were determined using Kruskal-Wallis and Dunn's multiple comparison test with a threshold for significance set to 0.05. Data not conducive to further specific statistical testing are presented as mean ± 95% confidence interval, adhering to the p < 0.05 criterion for statistical significance. All statistical analyses were conducted using GraphPad Prism 8 software (GraphPad Software Inc., San Diego, CA, USA).

## Results

### Characteristics of isolated EVs

**Fig. 1:**
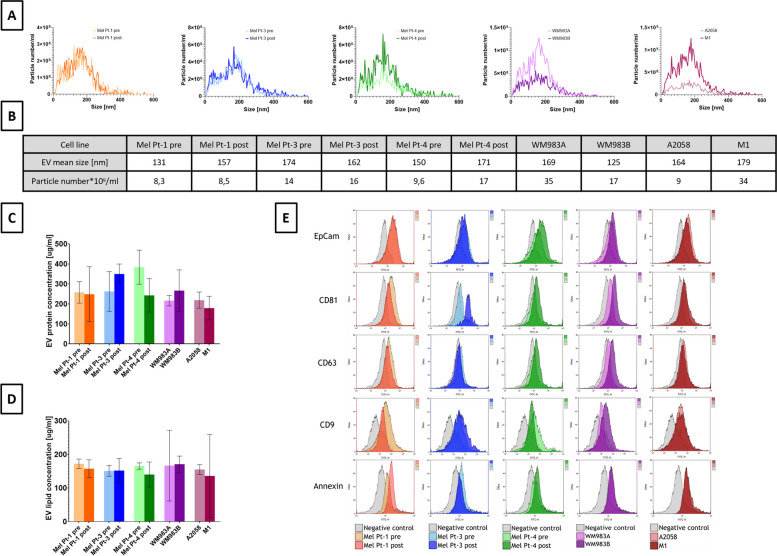
Characteristics of isolated extracellular vesicles. **A** Concentration and size distribution measured by Nanoparticle Tracking Analysis; **B** Mean size and particle number ×10^9^/ml isolate (SD≤10%); **C** EV sample protein concentration (μg/ml; mean ± 95% CI; **D** EV fraction lipid concentration (μg/ml; mean ± 95% CI); E) Flow cytometry analysis of EV markers with their isotype control latex-beads

To investigate EVs’ role in cancer progression, first EVs were isolated and characterized from the conditioned media of all five syngeneic pairs of cell lines. Nanoparticle tracking analysis (NTA) of the EV fractions confirmed their presence, with an average size ranging from 125 to 179 nanometers (Fig. 1A, B), consistent with previously reported values for EVs derived from supernatants [73]. The particle concentration ranged from 8,3x10^9^ to 35x10^9^/ml. We compared the lipid concentration and EV-associated protein content of each cell line, revealing no significant correlation with the respective aggressiveness levels (Fig. 1C, D). Flow cytometry confirmed the presence of commonly used extracellular vesicle markers (EpCam, CD81, CD63, CD9, Annexin) [74–76] (Fig. 1E) with the least positivity for CD63.

### Extracellular vesicles modify melanoma cells’ migratory capacity rather than proliferation

**Fig. 2:**
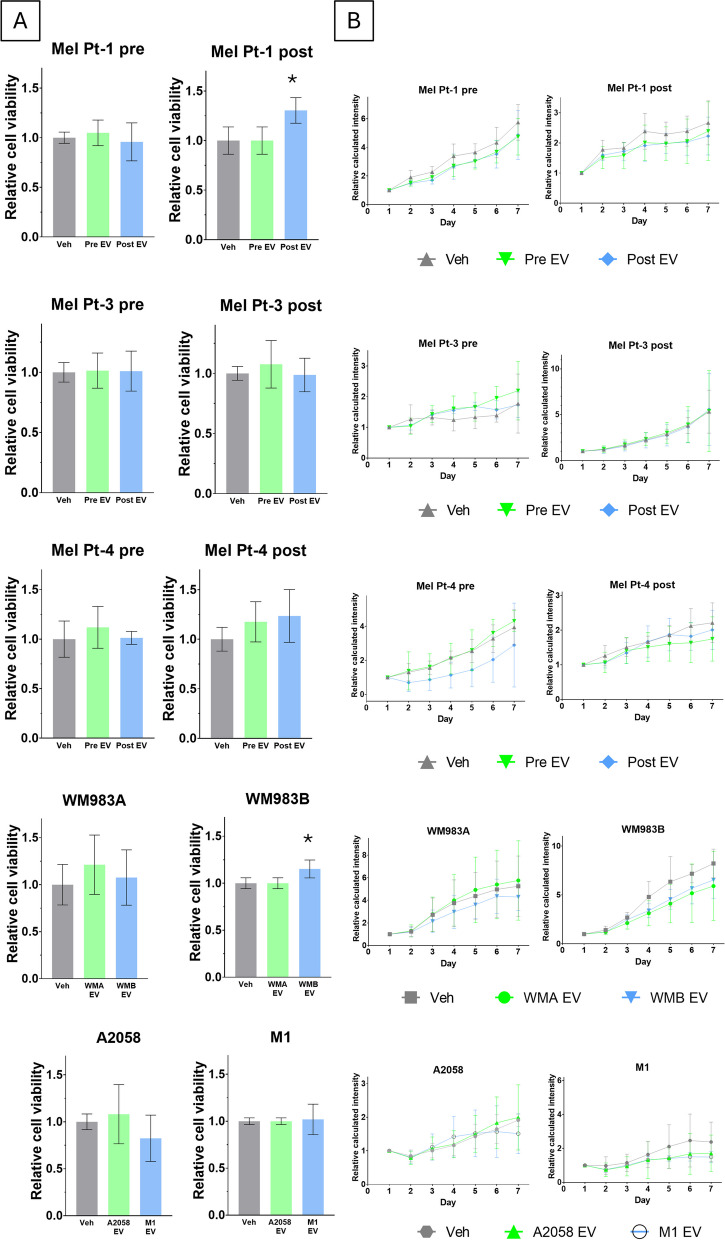
Extracellular vesicle’s effect on cell viability and sphere growth. Cells were treated with their own and pair-derived EV isolate (10 μg/ml protein content). **A** SRB assay: SRB staining was performed after 72 hours. Asterisks indicate significant differences to control using Kruskal-Wallis and Dunn's multiple comparison test. Results of three independent measurements are shown as mean ± SEM, and a p-value less than 0.05 was considered as statistically significant.; **B**) Spheroid formation assay: spheroid growth was monitored for 7 days. Spheroid size was calculated based on diameter, area, integrated density and median pixel intensity of the respective well and spheroids, the relative calculated signal intensity is shown as mean ± 95% CI (*n*=6)

The impact of EVs on cell viability was assessed using the Sulforhodamine B (SRB) assay. Mel Pt-1 post and WM983B cells treated with their own EVs showed a significant increase in cell viability, any other treatment with the more aggressive cell line-derived EV had only modest effect on cell viability (Fig. 2A). Similarly, treatments with the less-aggressive cells produced EVs had minimal effect on cell proliferation (Fig. 2A).

To further probe the tumorigenic potential of EVs within a more complex 3D tumor model, we conducted a spheroid growth assay (Fig. 2B). All the investigated cell lines formed spheroids 24 hours after seeding, thus all could be treated with their own and their pairs’ EVs. EV treatment resulted in only minor changes in spheroid growth. The effect of the treatment did not differ if cells were treated with EVs isolated from the supernatant originating from cells representing the more or less aggressive stage of the tumor (Fig. 2B).

**Fig. 3:**
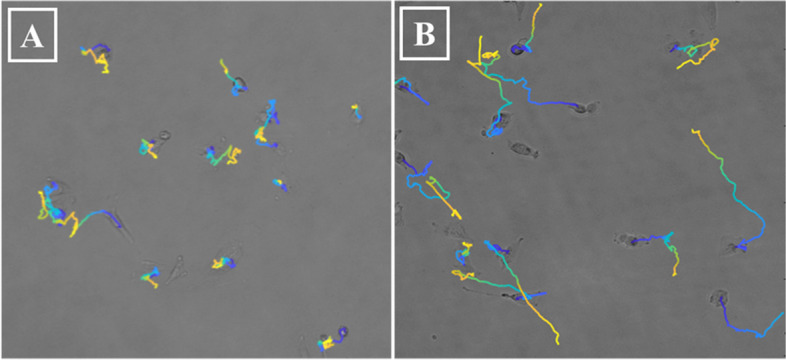
Trajectories of individual cells, drawn by marking the position of the cells and connecting these points during the whole recording. The color of the depicted trajectories refers to the time elapsed in the order of blue-green-yellow. **A** Vehicle control; **B** EV treated Mel Pt-4 post cells

**Fig. 4:**
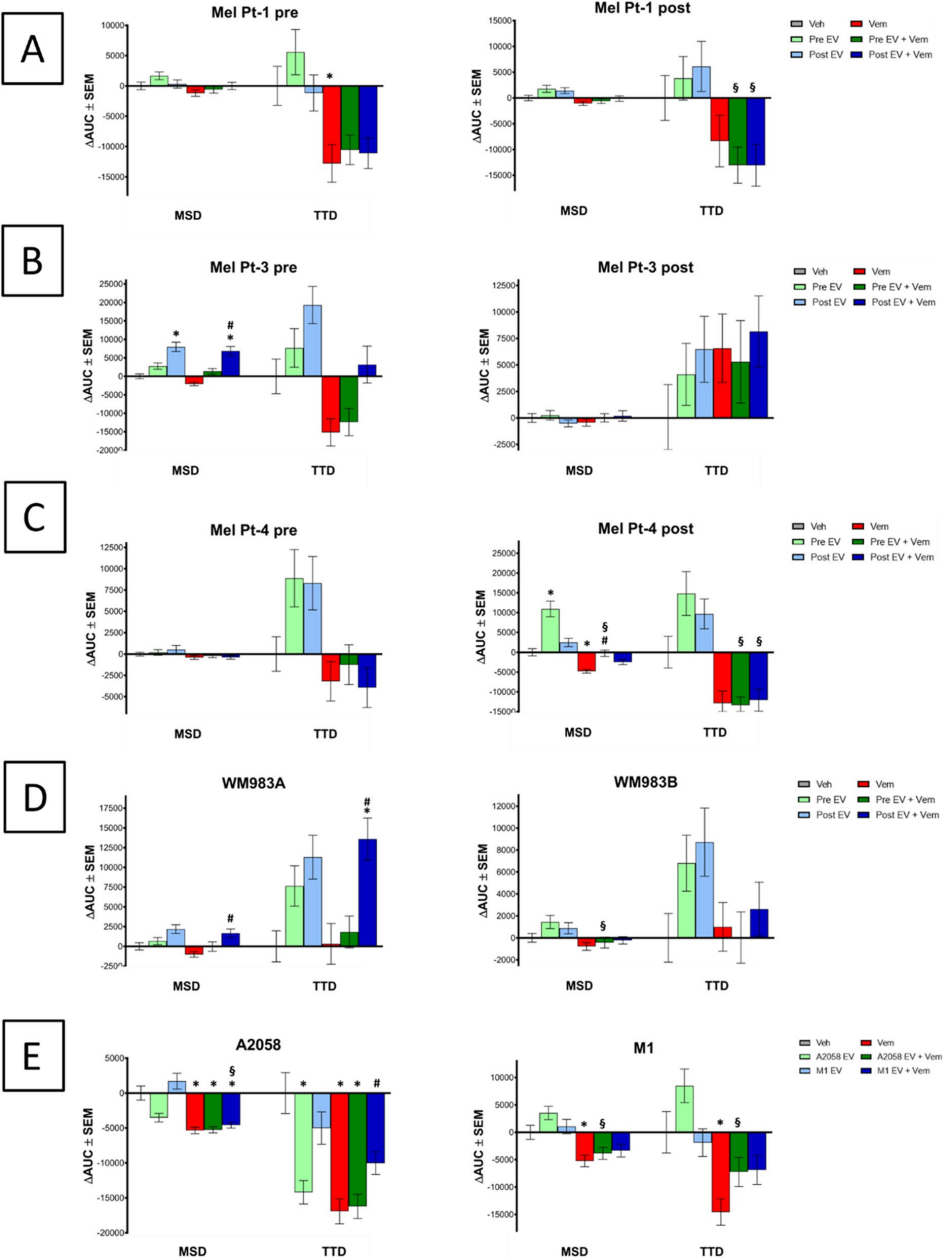
Effect of EV, vemurafenib (Vem) and combined (EV+Vem) treatment on single-cell migration. Cell migration was recorded for 24 hours, semiautomatic tracking of single cells performed using CellTracker and MSD and TTD calculated. Results of three independent measurements are shown as mean ± SEM, and p-value less than 0.05 considered as statistically significant (to vehicle: *, to Vem: #, to EV-only: §)

Video-microscopic measurements and subsequent single-cell tracking were performed to investigate a possible effect of EV treatment on cell motility (Fig. 3). Two different commonly used parameters: total travelled distance (TTD) and mean square displacement (MSD) were calculated to have a more detailed view of the changes in the migratory activity of the cells (Fig. 4). (MSD and TTD as a function of time is presented in Sup.Table1).

A cell line-dependent effect of EV treatment on cell migration was observed. Furthermore, the two calculated parameters did not exhibit congruent effects. In the case of Mel Pt-3 pair of cell lines, the pre cells’ migration was stimulated with both EVs, although a significant increase was only evident in MSD after the treatment with the post-cell-derived EVs (Fig. 4B). Conversely, for the Mel Pt-4 pre-cell line, only TTD appeared noticeably increased (Fig. 4C). Notably, while MSD of Mel Pt-4 post-cells significantly rose when treated with pre-EVs, the increase in TTD was not significant. Mel Pt-1 pre/post, Mel Pt-3 post, WM983A, WM983B and M1 cells did not show significant differences in any of the calculated parameters if treated with EVs alone. Nevertheless, WM983A/WM983B EV treatment led to a modest increase in MSD and in TTD (Fig. 4D). An increment in the migration of M1 cells was also observed following the treatment with A2058-derived EVs (Fig. 4E). Interestingly, A2058-derived cell line’s TTD was reduced as a reaction to EV treatment, however MSD was only decreased if the A2058 EVs were used for treatment. Altogether, there was no uniform difference between the migratory effect of less and more aggressive cell line-derived EVs.

### EV treatment could compensate migration inhibitory effect of vemurafenib

After observing that EVs showed a more distinct effect on migration, than on proliferation, the question if BRAF inhibiton could undermine the effect of EVs on migration was investigated. Since the Mel Pt post cell lines were established from patients, who were receiving already vemurafenib treatment the initial investigation was carried out using vemurafenib, as a BRAF inhibitor. Sensitivity of the cell lines to vemurafenib was determined using the SRB assay (SupFigS1). Sensitivity of A2058 and M1 did not exhibit significant differences, whereas WM983A cells displayed higher sensitivity compared to WM983B, its metastatic counterpart. The vemurafenib sensitivity of the Mel Pt-1 cell lines did not show notable differences. However, Mel Pt-3 pre cells were more susceptible to vemurafenib as compared to the corresponding post cell line. In contrast, in Mel Pt-4 cells, the observed vemurafenib sensitivity was higher in the post cells.

**Fig. 5:**
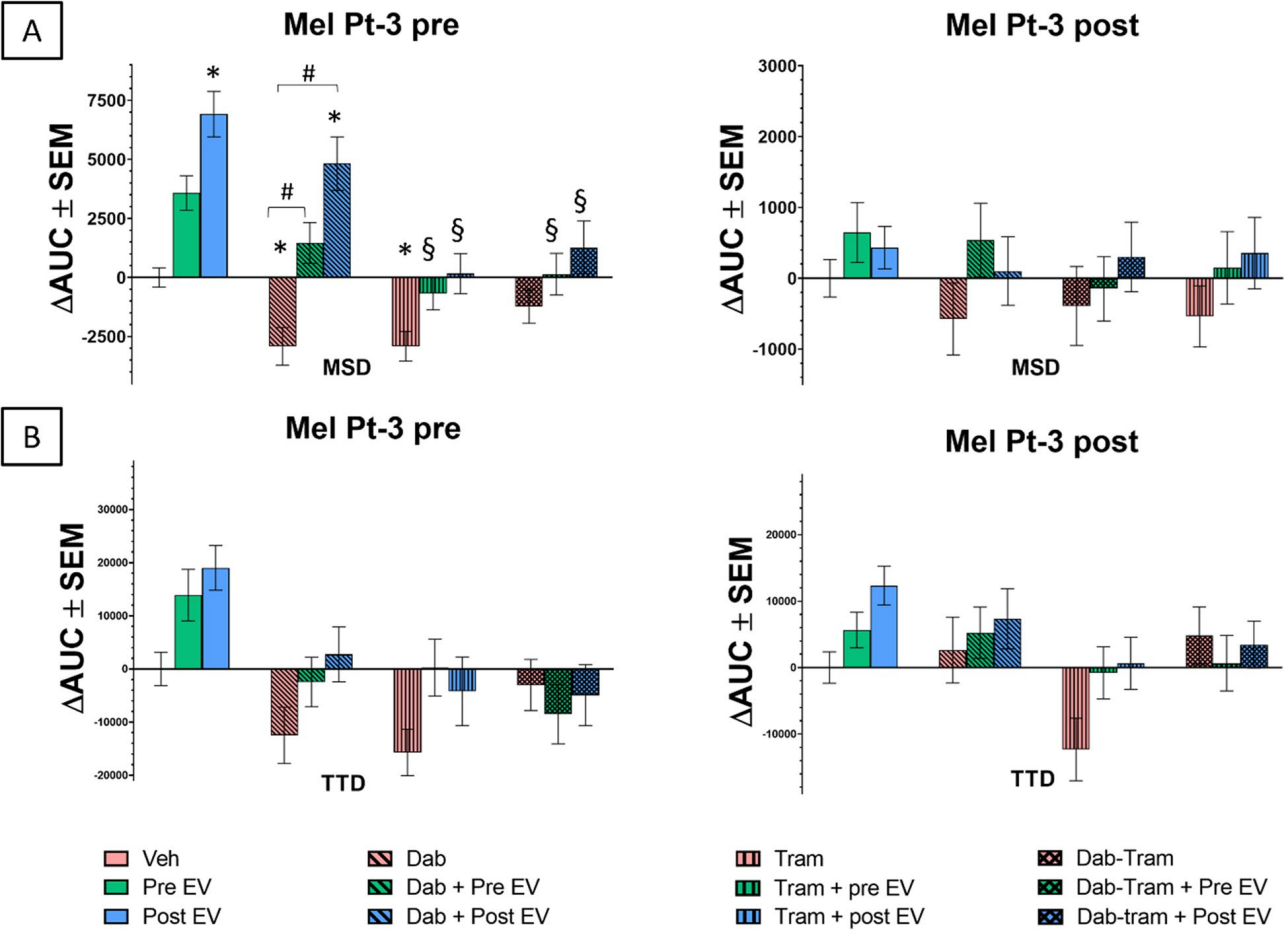
Effect of EV, Dabrafenib (Dab), Trametinib (Tram) and combined treatment on single-cell migration. Cell migration was recorded for 24 hours, semiautomatic tracking of single cells performed using CellTracker and MSD and TTD calculated. Results of three independent measurements are shown as mean ± SEM, and *p*-value less than 0.05 considered as statistically significant (to vehicle: *, to Dab: #, to EV-only: §)

Vemurafenib concentrations around the cells’ GI50 values were used for the video-microscopy experiments. The calculated ∆AUC values are shown in Fig. 5. (MSD and TTD as a function of time is presented SupTable2.) Vemurafenib markedly reduced cell migration quantified as MSD and TTD in most of the cell lines. Although, vemurafenib treatment did not show a migration inhibitory effect in TTD of WM983A, WM983B and Mel Pt-3 post cells. Of note, in Mel Pt-3 post cells MSD and TTD revealed opposite but not significant migratory effect.

Significantly higher MSD was measured in the more sensitive cells (Mel Pt-3 pre, Mel Pt-4 post and WM983A) uppon treatment with vemurafenib and EVs from the more resistant cells as compared to vemurafenib treatment. Albeit the same effect in TTD was significant only in WM983A cells. In combination with EVs, vemurafenib demonstrated the ability to attenuate the modest migration-promoting effect of EVs in Mel Pt-1 cells (Fig. 4A). In case of A2058 and M1 cells, EVs were unable to significantly counteract the migration-inhibitory effect of vemurafenib, with the exception observed in TTD of A2058 cells when treated with vemurafenib in combination with M1-derived EVs (Fig. 4E). In the more resistant cells (Mel Pt-3 post, Mel Pt-4 pre and WM983B) no significant difference in MSD and TTD was detected when treated with vemurafenib alone or in combination with EVs.

### EV treatment could compensate migration inhibitory effect of dabrafenib, but not trametinib or the combined treatment of dabrafenib and trametinib

Given that the Mel Pt cell lines were established during vemurafenib treatment, our initial investigation aimed to determine whether vemurafenib could counteract the effects of EVs. Upon observing that EVs attenuated the migration inhibitory effect of vemurafenib, we extended our inquiry to include dabrafenib, a more recently applied BRAF inhibitor. Seeing that EVs were effective against BRAF inhibitions, the treatments along the line with clinical practice were combined with the MEK inhibitor trametinib. We began by evaluating the sensitivity of the cell lines to dabrafenib, trametinib, and the dabrafenib-trametinib combination (SupFigS2,S3). Among the cell line pairs, Mel Pt-3 pre/post, Mel.

Pt-4 pre/post, and WM983A/WM983B, which displayed varying sensitivity to BRAF inhibition, were included in these assessments. Generally, cell lines representing less aggressive stages of the tumors (Mel Pt-3 pre, Mel Pt-4 pre, WM983A) exhibited greater sensitivity to dabrafenib or trametinib treatments. Combination treatments of dabrafenib and trametinib were conducted using three different concentrations, with combination indexes calculated for each experiment (SupFigS3). All, but one of the calculated combination indexes were below 1, indicating enhanced efficacy of the drug combination compared to individual treatments as previously observed [77]. The only exception was noted in the highest concentration of dabrafenib-treated WM983B, where a combination index exceeding 1 was discovered, indicating an antagonist effect. To investigate the EVs potential role in drug resistance the Mel Pt-3 cell line pair was used, since Mel Pt-3 pre/post cells sensitivity to vemurafenib, dabrafenib, trametinib and dabrafenib-trametinib differ greatly (SupFigS1,S3) and these cells have one of the the highest baseline migratory capacity (SupTable1).

Video-microscopy evaluation was performed as mentioned above and the calculated ∆AUC values are shown in Fig. 5. (MSD and TTD as a function of time are presented SupTable3.) In the case of Mel Pt-3 pre cells, treatment with dabrafenib, trametinib, and their combination led to reductions in both MSD and TTD parameters, although a significant decrease was only evident in the MSD values during single treatments. The MSD values measured in Mel Pt-3 post cells were also decreased compared to the vehicle control. Conversely, consistent with observations after vemurafenib treatment, a slight increase in TTD was observed when the treatment included a BRAF inhibitor.

In our experiments with Mel Pt-3 pre cell line both the pre and post cell-derived EVs could significantly compensate the inhibitory effect of dabrafenib on the MSD parameter. However, the EVs produced by the resistant cell line had a greater effect against the inhibition of dabrafenib. On the other hand, trametinib could diminish the effect of EVs, since the MSD values of the EV and trametinib-treated cells are significantly decreased compared to the treatment with EVs alone. While these effects were less equivocal in the TTD parameter. Furthermore, the migratory capacity of the Mel Pt-3 post cell line was modestly affected; however, a modest enhancement in motility was generally observed when these cells were treated in combination with EVs.

## Discussion

Extracellular vesicles are considered pivotal mediators in the intercellular communication network between both normal and tumor cells [78]. Given that metastasis poses a significant threat to the survival of cancer patients globally [23], there is considerable interest in elucidating the role of extracellular vesicles in cell migration and the initiation of metastatic processes [14].

The present study investigated the potentially different effects of EVs produced by cells – modelling less and more advanced stage tumors – with different sensitivity to BRAF (vemurafenib, dabrafenib) and MEK (trametinib) inhibitors. Five pairs of syngeneic melanoma cell lines were treated with EVs isolated from their or their pair’s supernatant. Characterization of isolated EVs and determination of their effects on cell proliferation and migration were performed. Characteristics of the EVs isolated from the supernatant of the cells by ultracentrifugation were relatable with the previously described supernatant-derived EV fractions [20, 78–80].

Cancer cell migration, as an essential process of metastasis formation, is widely studied. Since, the initial phase of the metastatic process involves invasion, during which tumor cells breach the basal membrane of their surrounding environment and traverse through the extracellular matrix (ECM) into adjacent tissues [81]. The effect of EV treatment on tumor cell migration is mostly investigated by transwell [13, 16, 20, 81–84] or wound healing assays [16, 85–89]. However, these assays are relatively complex and examine more aspects of the treatments’ effects simultaneously, like proliferation and invasion, not only migration. Moreover, these complex assays investigate cells’ migratory capacity in one direction (e.g. the closing of the scratch or migration through the membrane), overlooking the dynamic and multi-directional nature of cell migration [90, 91]. Single-cell tracking-based methods, as used in this study, are more direct approaches to investigate the treatments’ effect on cell migration. Two parameters MSD and TTD were calculated to quantify cell migration [71, 72]. Generally, greater effects can be observed in MSD if the treatment impacts the directionality of the movement to a higher extent than the velocity. In an earlier study, video-microscopy recording of scratches, and calculation of velocity could not prove the migration-promoting effect of melanoma-derived EVs in cancer-associated fibroblasts [92]. Of note, performing scratch assays limits the directionality of the cell migration as cells can migrate only in the direction of the induced space. Hence, a possible migrational effect of the treatment impacting the directionality of the migration is more challenging to detect.

In our results, for most of the cell lines, MSD and TTD values were elevated after EV treatments although a significant increase was only observed in the MSD parameter of the Mel Pt-3 pre and Mel Pt-4 post cell lines. However, it is noteworthy that EVs derived from more aggressive cell lines did not consistently exhibit an unequivocally greater effect. Given the critical role of cell migration in the initiation of metastasis, our findings lead us to propose that EVs play a more pronounced role in the process of metastasis formation rather than in tumor growth. These results are in line with earlier findings that oral squamous carcinoma cells treated with cancer-associated fibroblasts derived EVs showed increased migration to a greater extent than proliferation [93]. Likewise, EVs originating from cell lines with varying degrees of aggressiveness demonstrated no discernible impact on proliferation, nevertheless, EVs derived from more aggressive cells could enhance the migratory capacity of the recipient cells [13].

BRAF inhibition therapies (e.g. vemurafenib, dabrafenib), are widely used in clinics, however after the first promising period of the treatment, resistance can occurre in many patients [34, 35], that is often manifested in the acceleration of metastasis formation [50–52]. BRAF targeted therapy resistance can occur through multiple mechanisms, including EV-mediated intracellular cross-talks [48, 56, 94]. Our investigation unveiled the migration-promoting role of EVs, prompting us to explore whether BRAF inhibitors could reverse these elevated metastatic potentials. Our experimental findings demonstrated that EVs could attenuate the migration-inhibitory effects of BRAF inhibitors. Moreover, EVs derived from more resistant cells exhibited a more pronounced ability to counteract this inhibition. This observation aligns with previous studies indicating that EVs originating from V600E BRAF mutant PLX-4720-resistant cells transmit resistance to recipient cells by transporting PDGFRβ proteins [48]. Conversely, EVs from sensitive parental cells failed to increase proliferation [48]. Additionally, vemurafenib-resistant melanoma cells-derived EVs could increase the proliferation of sensitive cells during vemurafenib therapy compared to the control cells via transporting an ALK isoform (truncated ALK fused with murine leukemia virus (MMLV)) [56]. Furthermore, different BRAF splicing variants could be traced in EVs from resistant cell lines and plasma samples from relapsed patients treated with BRAF inhibitors [95]. In line with this, in our experiments, EVs from the more resistant cell lines could transmit resistance more effectively. In addition, earlier studies have shown that plasma EV-associated miRNAs in MM patients are linked to susceptibility towards MAPKi treatment, suggesting alterations in EV cargo in cases of resistance [96], a phenomenon also observed in our *in vitro* experiments.

Since BRAF inhibitor therapy is often combined with MEK inhibitors [58–60], we investigated if the combination treatment could diminish the migration-promoting effect of EVs. Nevertheless, if the cells (originated from patients under vemurafenib therapy [65]) were subjected to treatment with trametinib alone or in combination with dabrafenib, the EVs did not elicit the same response observed with individual BRAF inhibitor treatment. Therefore, a combined therapeutic approach utilizing both BRAF and MEK inhibitors (BRAFi/MEKi) holds promise in mitigating the migratory impact exerted by EVs. It is worth noting that resistance to BRAF inhibition may coincide with sustained phosphorylation of MEK, as evidenced by the relatively heightened levels of pMEK and pERK observed in post cells [65]. A notable limitation of the study is that these cells were derived from patients undergoing BRAF inhibition therapy and had not yet acclimated to the effects of MEK inhibitors. Nevertheless, our findings imply the possibility of propagating BRAF inhibitor resistance through EVs.

## Conclusions

In summary, our findings indicate that extracellular vesicles (EVs) derived from melanoma cells exerted a limited effect on cancer cell proliferation, while significantly promoting cell migration. Notably, EVs were found to counteract the migration-inhibitory effects of BRAF inhibitors, and EVs from more resistant cell lines exhibiting a more pronounced effect during vemurafenib and dabrafenib treatment. However, the combination of dabrafenib with trametinib was observed to attenuate the influence of EVs. Our results support previous findings indicating the crucial involvement of EVs in melanoma progression, particularly emphasizing their greater contribution to metastasis formation compared to primary tumor growth, while also highlighting their implication in BRAF inhibitor resistance.

### Supplementary Information

Supplementary Material 1. 

## Data Availability

Not applicable.
