# Supplementary material for: Extracellular vesicles promote migration despite BRAF inhibitor treatment in malignant melanoma cells
Source: Cell Commun Signal. 2024 May 22;22:282. doi: 10.1186/s12964-024-01660-4 (PMC11110207; doi:10.1186/s12964-024-01660-4)
Supplement: Supplementary file 1 — Supplementary Material 1. [file 12964_2024_1660_MOESM1_ESM.docx]

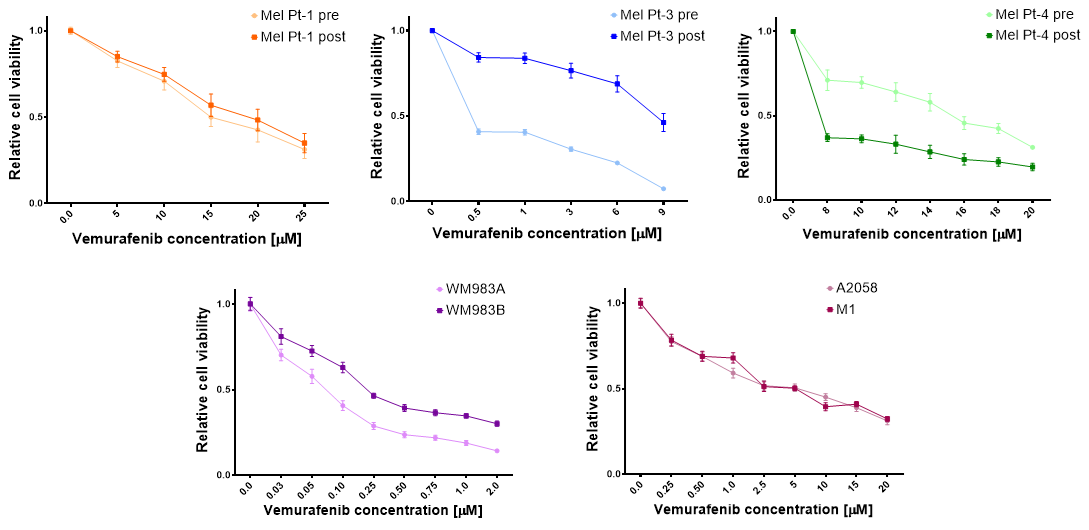


Figure S1. Cell viability relative to control following 72h vemurafenib treatment determined by SRB cell viability assay. Results of three independent measurements are shown as mean ± 95% CI.


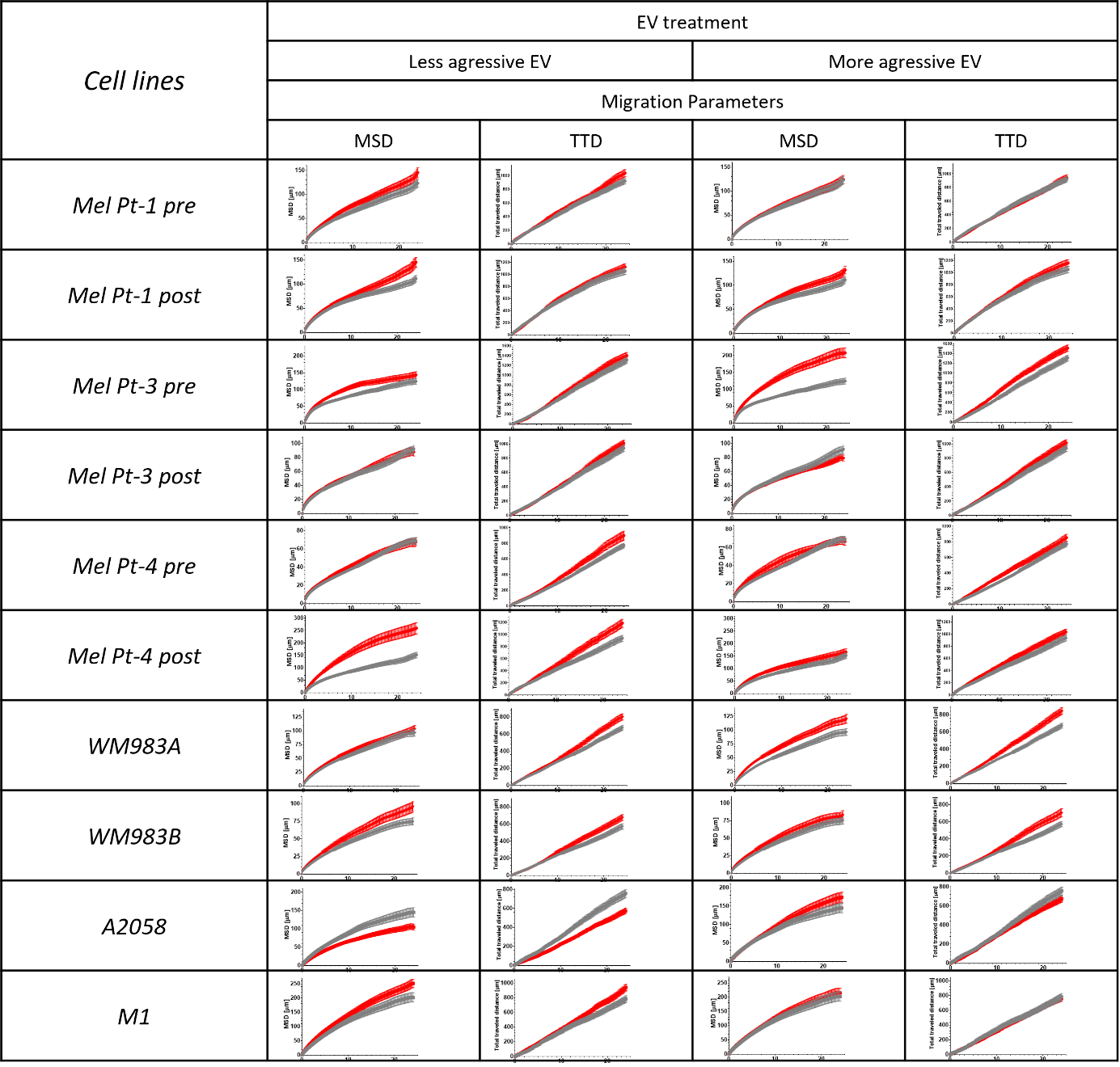


Table S1. Effects of EV single treatment on cell migration. Mean square displacement (MSD) and total travelled distance (TTD) as a function of time determined using 24h video-microscopy recording and semiautomatic cell-tracking with CellTracker. Results of three independent measurements are shown as mean ± SEM. Grey indicates the vehicle treatment and red represent the EV-treated cells.


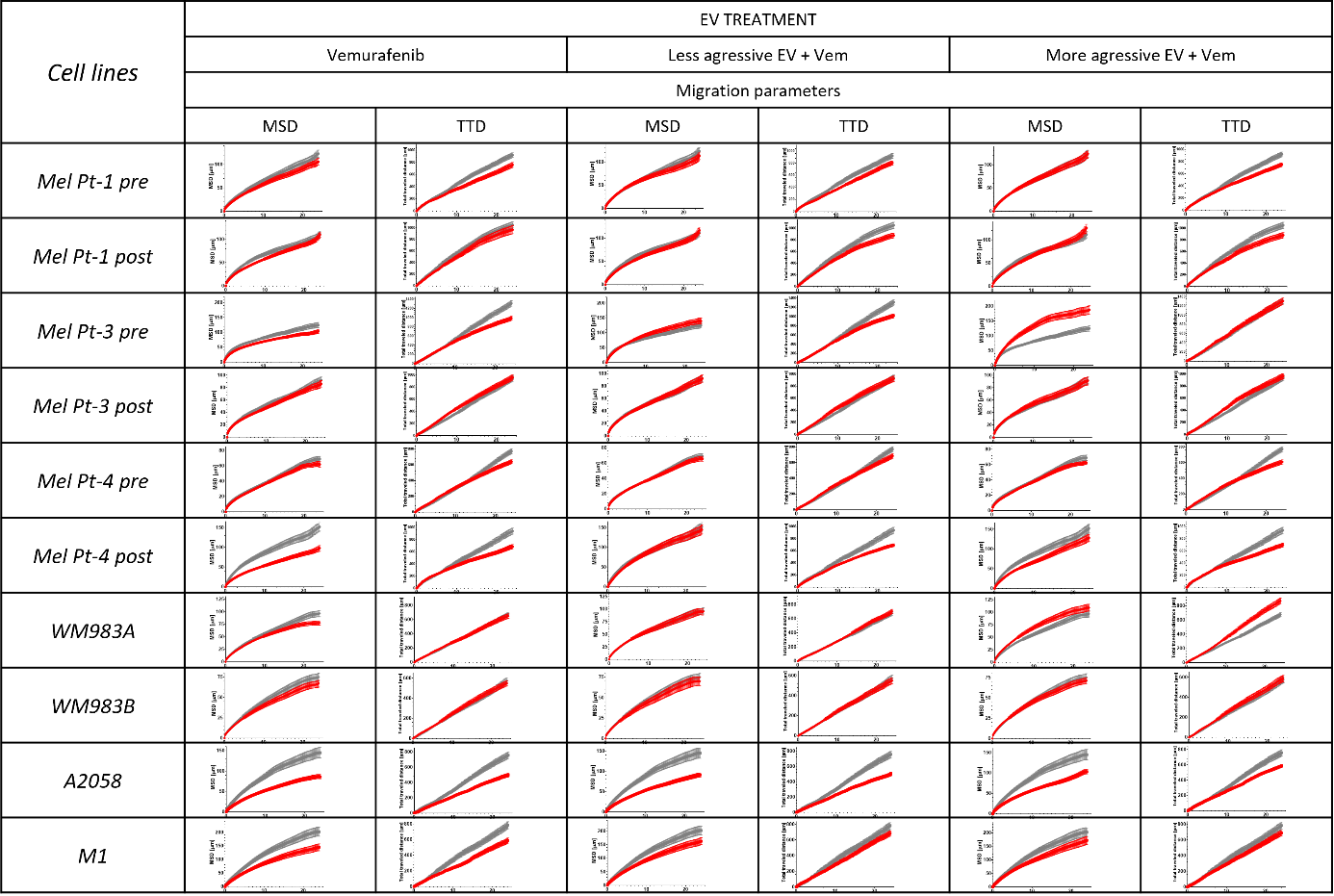


Table S2. Effects of EV and vemurafenib treatment on cell migration. Mean square displacement (MSD) and total travelled distance (TTD) as a function of time determined using

24h video-microscopy recording and semiautomatic cell-tracking with CellTracker. Results of three independent measurements are shown as mean ± SEM. Grey indicates the vehicle treatment and red represent the Vem/EV+Vem -treated cells.


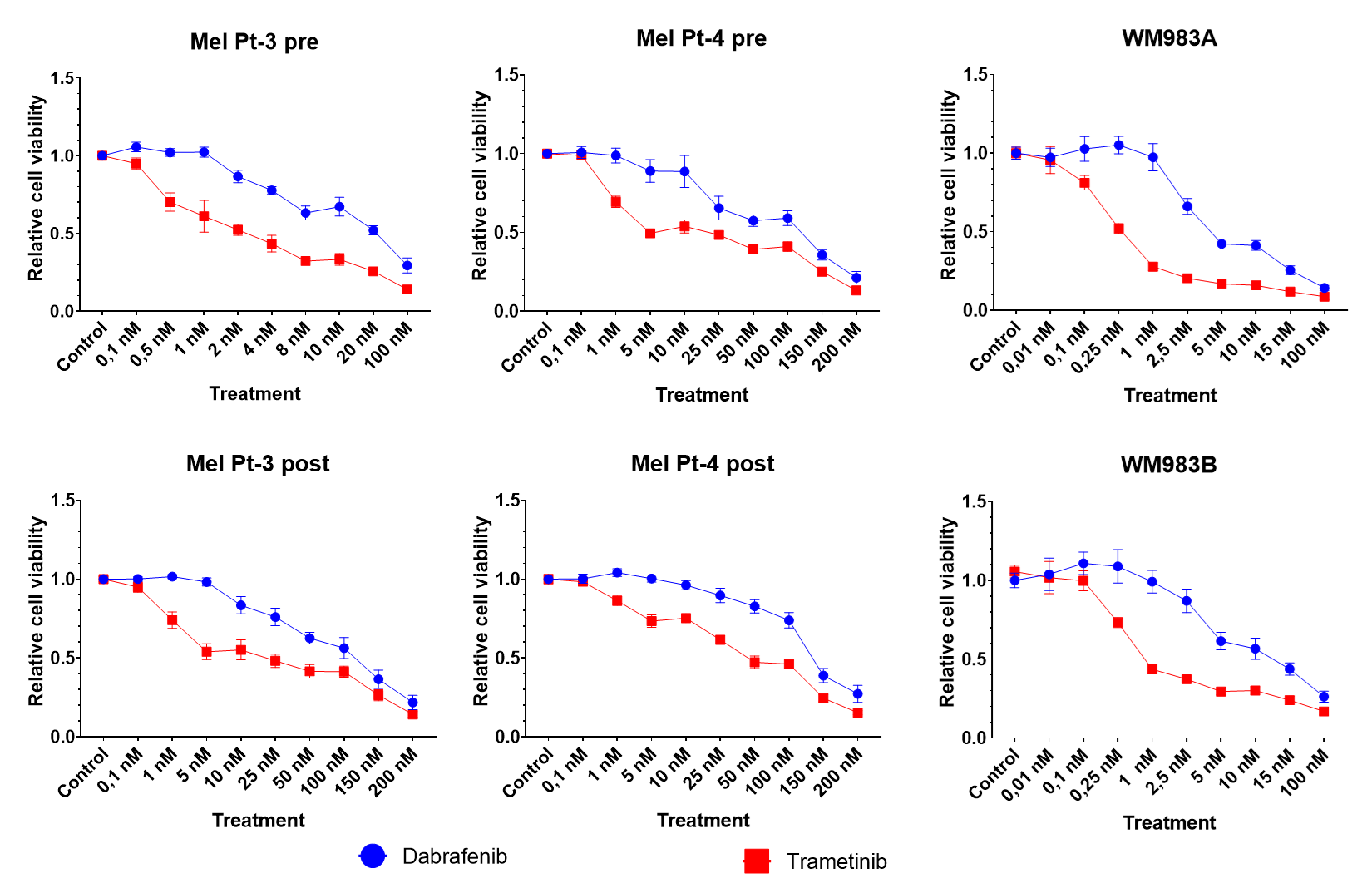


Figure S2: Cell viability relative to control following 72h dabrafenib or trametinib treatment determined by SRB cell viability assay. Results of three independent measurements are shown as mean ± 95% CI.


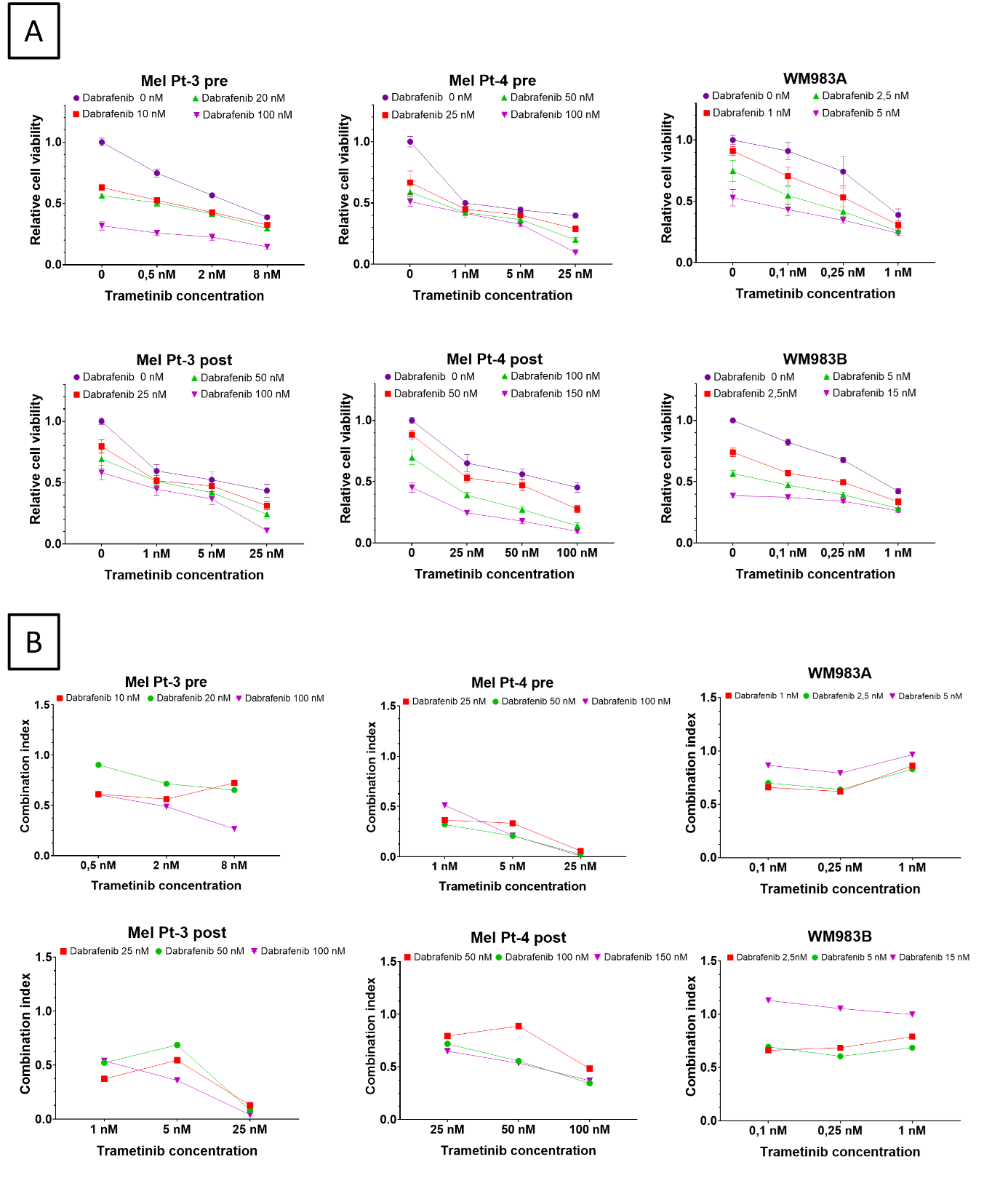


*Figure S3: The effect of 72 hours dabrafenib-trametinib combination treatment on cell viability determined by SRB cell viability assay. A) Cell viability relative to control in case of different dabrafenib-trametinib combinations. Results of three independent measurements are shown as mean ± 95% CI.; B) The combination indexes for the different dabrafenib-trametinib treatments. CI<1 synergism, CI=1 additive effect, CI>1 antagonism.*

*
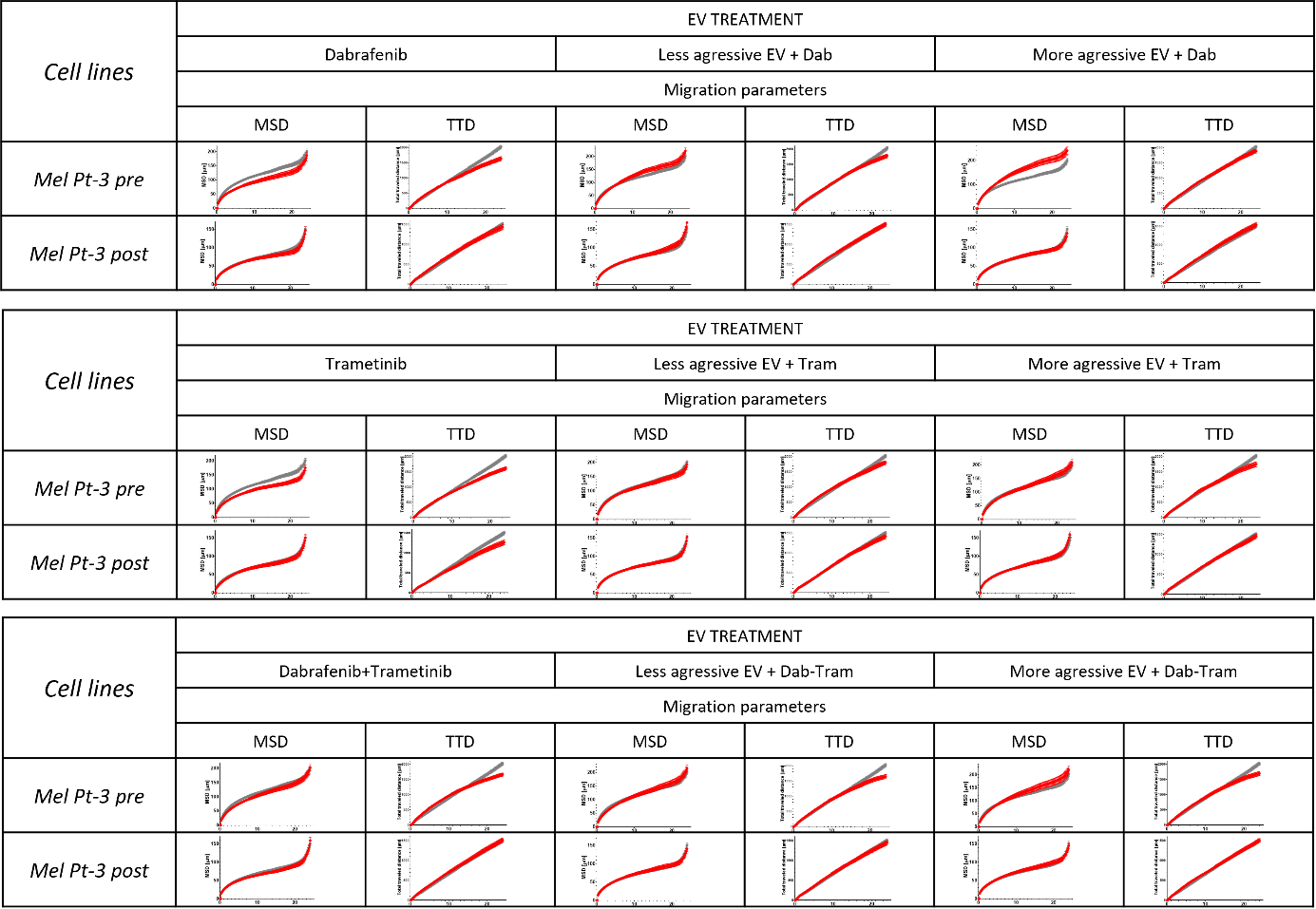
*

Table S3. Effects of EV and dabrafenib/trametinib/dabrafenib-trametinib treatment on cell migration. Mean square displacement (MSD) and total travelled distance (TTD) as a function of time determined using 24h video-microscopy recording and semiautomatic cell-tracking with CellTracker. Results of three independent measurements are shown as mean ± SEM. Grey indicates the vehicle treatment and red represent the Dabr/Tram/Dabr-Tram/Dabr+EV/Tram+EV/Dabr-Tram+EV -treated cells.
